# Rapid systematic review of smoking cessation interventions for people who smoke and have cancer

**DOI:** 10.1093/eurpub/ckac129.556

**Published:** 2022-10-25

**Authors:** K Frazer, N Bhardwaj, P Fox, V Niranjan, S Quinn, C Kelleher, P Fitzpatrick

**Affiliations:** 1 Nursing, Midwifery and Health Systems, University College Dublin, Dublin, Ireland; 2 Public Health, Physiotherapy, Sports Science, University College Dublin, Dublin, Ireland; 3 Department Preventive Medicine and Health Promotion, St Vincent's University Hospital, Dublin, Ireland

## Abstract

**Background:**

Higher rates of cancer are reported in smokers compared to non-smokers, and continued smoking following a cancer diagnosis is associated with reduced health outcomes and survival. Despite international evidence of increased risks, a substantial percentage of people with a cancer diagnosis continue to smoke. Patients may be unaware of the additional risks associated with continued smoking, and health care professionals may not engage with quit supports. As part of a larger feasibility study to develop a smoking cessation pathway in cancer services in Ireland, a rapid review of the evidence was completed.

**Methods:**

Systematic searches of PubMed, Embase, and CINAHL 2015 to December 2020 were conducted; with studies restricted to adults with a cancer diagnosis [lung, breast, cervical, head and neck] and published in English. No restriction was placed on study designs. 6404 studies were identified and uploaded into COVIDENCE platform, Cochrane's systematic review methods were adopted throughout, PRISMA reporting guidelines were used, and narrative data synthesis was completed (CRD 42020214204).

**Results:**

The twenty-three-studies report evidence from USA, Canada, England, Lebanon, and Australia. The setting for all interventions was hospitals and cancer clinics. Evidence identifies high dropout rates, inconsistencies in approaches and duration of smoking cessation interventions with varied outcomes. A wide-ranging number of critical components emerged associated with optimal quit support- including the timing of and frequency of quit conversations, use of electronic records, in-person support meetings, provision of nicotine replacement therapy and extended use of Varenicline, smoking cessation services embedded in oncology depts, and engaging with families wanting to quit at the same time.

**Conclusions:**

Developing tailored smoking cessation interventions are needed for smokers diagnosed with cancer to enable engagement.

**Key messages:**

• Continued smoking following a cancer diagnosis is associated with reduced health outcomes.

• Smoking cessation programmes for cancer patient should be tailored to meet needs.

